# Enhanced Catalytic Soot Oxidation over Co-Based Metal Oxides: Effects of Transition Metal Doping

**DOI:** 10.3390/molecules29010041

**Published:** 2023-12-20

**Authors:** Jianbin Luo, Xinbo Zhu, Zhiwei Zhong, Geng Chen, Yu Hong, Zijian Zhou

**Affiliations:** 1 Faculty of Maritime and Transportation, Ningbo University, Ningbo 315211, China; ljb15667840050a@163.com (J.L.); zhongzhiwei0302@163.com (Z.Z.); chengeng@nbu.edu.cn (G.C.); 2 New Materials Institute, University of Nottingham Ningbo China, Ningbo 315100, China; 3 State Key Laboratory of Coal Combustion, Huazhong University of Science and Technology, Wuhan 430074, China; zhouzj2012@hust.edu.cn

**Keywords:** soot oxidation, Co-based catalyst, NO, real soot, contact mode

## Abstract

A series of Co-M (M = Fe, Cr, and Mn) catalysts were synthesized by the sol-gel method for soot oxidation in a loose contact mode. The Co-Fe catalyst exhibited the best catalytic activity among the tested samples, with the characteristic temperatures (T_10_, T_50_, and T_90_) of 470 °C, 557 °C, and 602 °C, respectively, which were 57 °C, 51 °C, and 51 °C lower than those of the CoO*_x_* catalyst. Catalyst characterizations of N_2_ adsorption–desorption, X-ray diffraction (XRD), X-ray photo-electron spectrometry (XPS), and the temperature programmed desorption of O_2_ (O_2_-TPD) were performed to gain insights into the relationships between the activity of catalytic soot oxidation and the catalyst properties. The content of Co^2+^ (68.6%) increased due to the interactions between Co and Fe, while the redox properties and the relative concentration of surface oxygen adsorption (51.7%) were all improved, which could significantly boost the activity of catalytic soot oxidation. The effects of NO and contact mode on soot oxidation were investigated over the Co-Fe catalyst. The addition of 1000 ppm of NO led to significant reductions in T_10_, T_50_, and T_90_ by 92 °C, 106 °C, and 104 °C, respectively, compared to the case without the NO addition. In the tight contact mode, the soot oxidation was accelerated over the Co-Fe catalyst, resulting in 46 °C, 50 °C, and 50 °C reductions in T_10_, T_50_, and T_90_ compared to the loose contact mode. The comparison between real soot and model Printex-U showed that the T_50_ value of real soot (455 °C) was 102 °C lower than the model Printex-U soot.

## 1. Introduction

Diesel engines are widely applied in transportation and industries due to their excellent economic effectiveness, performance, and stability [1,2]. However, the emissions of a substantial amount of soot particles from diesel engines cause severe air pollution and even pose significant threats to the respiratory and cardiovascular systems of human beings. Particles were classified as the main object of environmental governance by the governments around the world. The United States environmental protection agency (EPA) and the Euro VI regulatory standard imposed stringent limits on particles emissions, while emphasizing the installation of diesel particulate filters (DPFs) in vehicle exhaust systems as a key measure to control particle emissions [3,4]. Diesel particulate filters (DPFs) are widely implemented for soot capture regarding diesel engines. However, DPF instruments become clogged by soot particles after long-term operations [5]. Currently, catalyzed diesel particulate filters (CDPFs) are regarded as an emerging technology for extending the lifetime of DPFs since the captured soot can be oxidized at high temperatures in the presence of a catalyst [6].

In recent years, mixed-metal oxides have been widely used for the catalytic oxidation of soot, CO, and volatile organic compounds due to their comparable catalytic activity and high thermal and chemical stabilities compared to single-component oxides [7,8]. Kuwahara et al. reported that the doping of Fe into Mn_2_O_3_ significantly increased the concentration of adsorbed oxygen, resulting in a decrease in the T_50_ of the MnO*_x_* catalyst from 339 °C to 328 °C for soot oxidation [9]. Doggali et al. reported that the Mn-ZrO_2_ catalyst-initiated CO oxidation at 80 °C, while CO oxidation started at 160 °C over the other three catalysts of Cu-ZrO_2_, Fe-ZrO_2_, and Ni-ZrO_2_ [10]. Ali et al. reported that the introduction of transition metals could increase the amount of oxygen vacancies on the surface of Cu-Mn-Ce, consequently promoting soot oxidation, as a remarkable reduction in T_50_ was observed over the optimized Cu_1_Mn_1_Ce_1_ catalyst by around 100 °C when compared to Pt/Al_2_O_3_ [11]. Li et al. found that T_50_ slightly decreased by 15.6 °C over the Fe_10_Ce_90_ catalyst when compared with the CeO_2_ catalyst, which was due to the introduction of Fe that enhanced the content of Ce^3+^, while the Ce^3+^ content was closely related to oxygen vacancies [12]. Feng et al. discovered that K/La_0.8_Ce_0.2_Mn_1−*x*_Fe*_x_*O_3_ exhibited higher catalytic activity compared to the La_0.8_Ce_0.2_Mn_1−*x*_Fe*_x_*O_3_ catalyst, while the presence of K ions as electron donors promoted the adsorption and dissociation of gaseous oxygen on the catalyst’s surface [13].

Co-based catalysts have been extensively investigated in catalytic soot oxidation studies due to their excellent thermal stability, comparable activity, and relative low cost [14,15]. Zou et al. reported that Co_0.93_Ce_0.07_O*_x_* catalysts exhibited lower T_90_ temperatures (407 °C) in the catalytic soot oxidation process than Co_3_O_4_ catalysts (437 °C) in a loose contact mode since the interactions between Co and Ce could promote the generation of oxygen vacancies on the catalyst’s surface [16]. Tsai et al. found that the value of T_90_ for a three-dimensional nanostructured Co_3_O_4_ catalyst was 448 °C, approximately 42 °C lower than conventional Co_3_O_4_ nanoparticles [17]. J. C. Medina et al. reported that the value of T_50_ over the CeO_2_/Co_3_O_4_ catalyst decreased by roughly 46 °C compared to pure CeO_2_ due to the excellent dispersion of Co_3_O_4_ crystals in the Ce-based microstructure [18]. Zhang et al. stated that the partial replacement of cobalt by Ni in a CoAl oxide increased the number of oxygen vacancies on the surface of catalyst; thus, the value of T_50_ over the Co_1.5_Ni_0.5_AlO catalyst was 57 °C lower than that of the Co_3_O_4_ catalyst. Yi et al. reported that T_50_ slightly decreased by 68 °C over the Ag/Co_3_O_4_ catalyst when compared with the Co_3_O_4_ catalyst, which was due to the interactions between Ag and Co_3_O_4_ that improved the generation of active oxygen species, while Ag could enhance the number of oxygen vacancies [19]. Gao et al. reported that the Ce partially replaced by Co and Fe elements over the Ce-Co-Fe catalyst could induce the formation of oxygen vacancies for increasing the surface area and active sites [20]. However, to the best of our knowledge, there have been limited reports on catalytic soot oxidation using Co-based catalysts doped with different transition metals. Furthermore, the potential mechanisms of catalytic soot oxidation in terms of the contact mode and NO addition over Co-based catalysts remain unclear [21].

In this work, Co-M (M = Fe, Cr, and Mn) catalysts are prepared using the sol-gel method for soot oxidation and compared with a pure Co_3_O_4_ catalyst. The effects of NO addition and the contact mode between the catalyst and soot particles on catalytic soot oxidation are also studied. Furthermore, the activity of the catalytic oxidation of real soot and Printex-U are compared. The structure and chemical properties of the Co-M catalysts are investigated using N_2_ adsorption–desorption, X-ray powder diffraction (XRD), X-ray photoelectron spectroscopy (XPS), and temperature-programmed oxidation (O_2_-TPD) methods. The reaction mechanisms of soot oxidation over Co-M catalysts are also proposed.

## 2. Results and Discussion

### 2.1. Textural Properties

**Figure 1:**
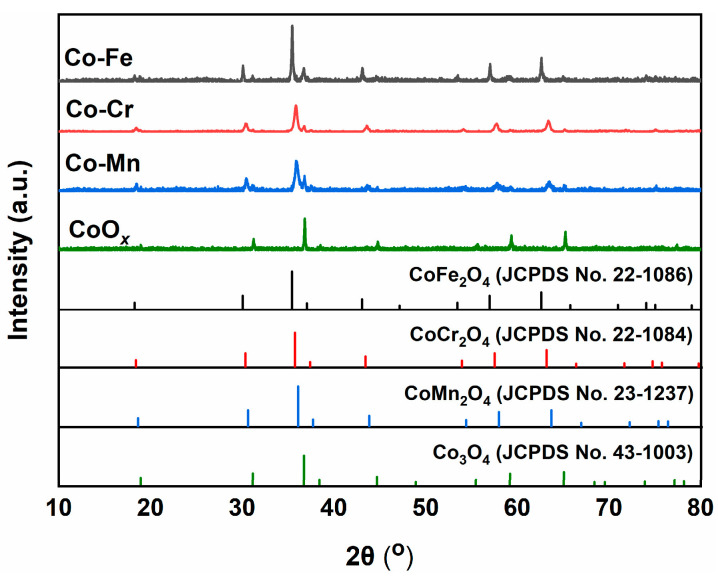
XRD patterns of the Co-M (M = Fe, Cr, and Mn) catalysts.

Figure 1 shows the XRD patterns of the Co-M (M = Fe, Cr, and Mn) catalysts. Overall, all Co-M catalysts exhibited sharp and intense diffraction peaks, indicating that the catalysts had a well-crystallized structure after high-temperature calcination at 600 °C. The diffraction peaks of the CoO*_x_* catalyst at 2θ values of 19°, 36.8°, 55.6°, and 59.3° matched well with the (1 1 1), (3 1 1), (4 2 2), and (5 1 1) planes of crystalline cubic fluorite Co_3_O_4_ (JCPDS No. 43-1003), respectively [22]. For the Co-Fe catalyst, the diffraction peaks at the 2θ values of 18.2°, 35.4°, 56.9°, and 62.5° were assigned to the (1 1 1), (3 1 1), (5 1 1), and (4 4 0) planes of the cubic CoFe_2_O_4_ crystal (JCPDS No. 22-1086), respectively [23]. For the Co-Cr catalyst, the diffraction peaks at the 2θ values of 18.3°, 35.7°, 57.4°, and 63.1° were assigned to the (1 1 1), (3 1 1), (5 1 1), and (4 4 0) planes of the cubic CoFe_2_O_4_ crystal (JCPDS No. 22-1084), respectively [24]. Furthermore, for the Co-Mn catalyst, the major diffraction peaks centered at the 2θ values of 18.5° (1 1 1), 57.9° (3 1 1), 35.9° (5 1 1), and 63.6° (4 4 0) corresponded to the cubic MnCo_2_O_4_ (JCPDS No. 23-1237) [25].

**Figure 2:**
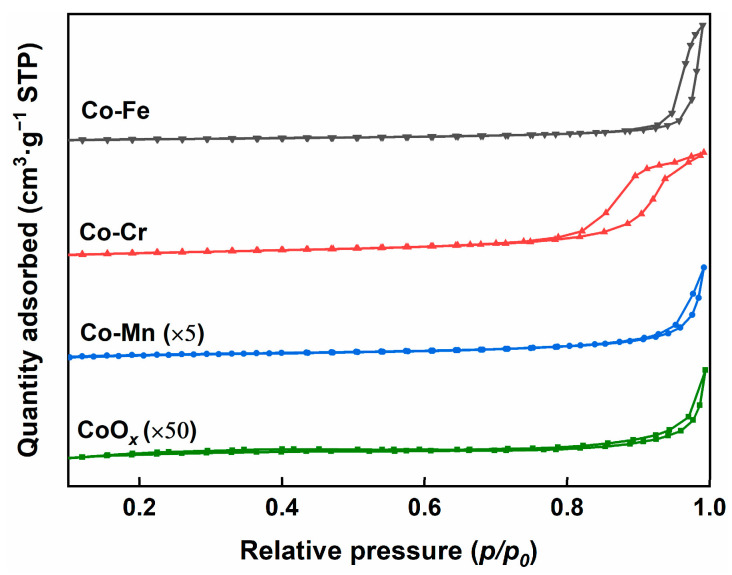
N_2_ adsorption–desorption isotherms of the Co-M (M = Fe, Cr, and Mn) catalysts.

Figure 2 shows that the Co-M (M = Fe, Cr, and Mn) catalysts possess type-IV isotherms, indicating that the pore structures of the samples are primarily mesoporous. The BET surface area, pore volume, and average pore diameter of the Co-based catalysts (Co-M, M = Fe, Cr, and Mn) are shown in Table 1. The Co-Cr catalyst has the highest specific surface area of 18.9 m^2^·g^−1^, followed by Co-Fe (9.9 m^2^·g^−1^), Co-Mn (3.7 m^2^·g^−1^), and CoO*_x_* (0.5 m^2^·g^−1^). The relevant studies indicated that an increase in the specific surface area of the catalyst could promote contact between the soot particles and catalyst, therefore enhancing soot oxidation [26]. The pore volumes and average pore diameters of the Co-M (M = Fe, Cr, and Mn) catalysts are higher than that of CoO*_x_*. Specifically, the pore volumes for Co-Fe, Co-Cr, Co-Mn, and CoO*_x_* catalysts are 95.3 mm^3^·g^−1^, 89.1 mm^3^·g^−1^, 14.5 mm^3^·g^−1^, and 1.3 mm^3^·g^−1^, respectively. Additionally, the average pore diameter of the Co-Fe catalyst is 38.6 nm, significantly larger than those of the Co-Cr (18.9 nm), Co-Mn (15.7 nm), and CoO*_x_* (9.9 nm) catalysts. Lee et al. also discovered that the mesoporous Ce-based catalysts accelerated soot oxidation, which could be ascribed to the larger pore diameter of the catalyst, which enhanced the interaction between the catalyst and soot particles [27].

### 2.2. Redox Properties

**Figure 3:**
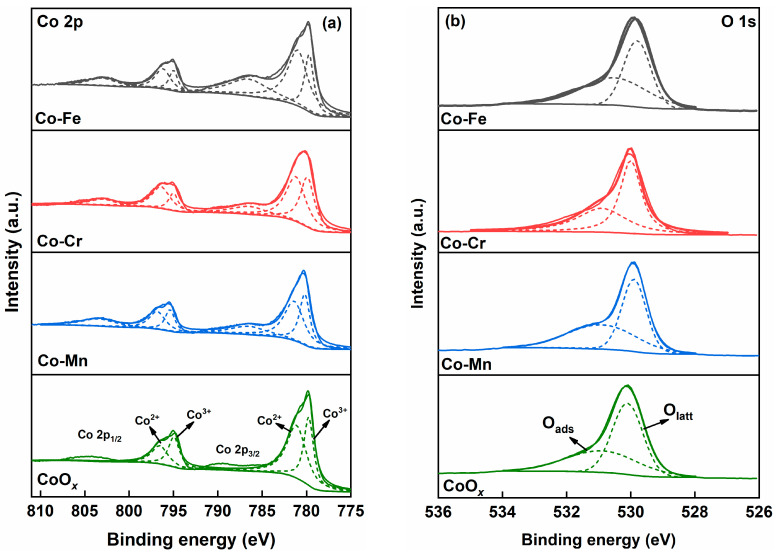
XPS spectra of the Co-M (M = Fe, Cr, and Mn) catalysts: (**a**) Co 2p and (**b**) O 1s.

XPS spectra were obtained to investigate the surface chemical compositions of the Co-M (M = Fe, Cr, and Mn) catalysts. As shown in Figure 3a, the peaks of Co 2p_3/2_ and Co 2p_1/2_ are around 780.0 and 795.0 eV [28]. Moreover, two peaks at 779.8 and 781.5 eV were observed after the deconvolution of the Co 2p_3/2_ orbits for the Co-M catalysts. The former peak could be ascribed to the Co^3+^ species, while the latter one belonged to the Co^2+^ species [29]. After the deconvolution of the Co 2p_1/2_ orbital, two peaks were observed at 794.6 and 796.5 eV, corresponding to the Co^3+^ and Co^2+^ species, respectively [30]. The relative concentration of Co^2+^ species was defined as Co^2+^/(Co^2+^ + Co^3+^). The Co-Fe catalyst possessed the highest relative Co^2+^ concentration of 68.6%, followed by Co-Cr (60.4%), Co-Mn (58.6%), and CoO*_x_* (56.6%).

The O 1s spectra of the Co-M (M = Fe, Cr, and Mn) catalysts could be fitted into two component peaks within 529.7 to 531.5 eV and 532.7 to 533.5 eV (shown in Figure 3b), which corresponded to lattice oxygen (O_latt_) and surface adsorbed oxygen (O_ads_) species, respectively [31]. Table 2 shows the relative concentration of O_ads_ over the Co-M catalysts, defined as O_ads_/(O_ads_ + O_latt_). The values were in the order of Co-Fe (51.7%) > CoO*_x_* (47.9%) > Co-Mn (42.0%) > Co-Cr (31.2%). Previous studies confirmed that surface adsorbed oxygen species demonstrated better mobility and reactivity compared to lattice oxygen to catalytic soot oxidation in a loose contact mode [32]. Shang et al. found that doping Bi metal into the Co_3_O_4_ catalyst boosted the amount of adsorbed oxygen from 0.63 × 10^−4^ mol·g^−1^ to 4.55 × 10^−4^ mol·g^−1^, resulting in a decrease in the T_50_ value of Bi_0.2_Co_0.8_O*_x_* by 102 °C [33].

**Figure 4:**
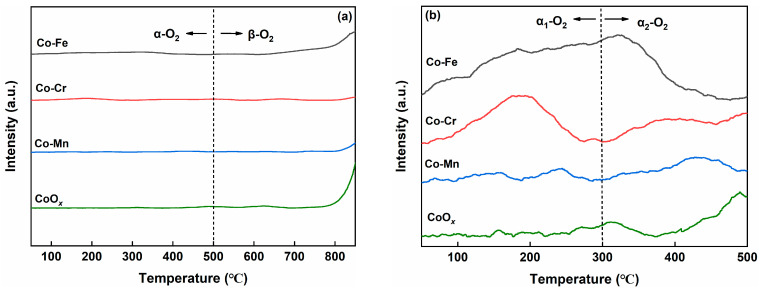
O_2_-TPD profiles (**a**) and enlarged O_2_-TPD profiles from 50 °C to 500 °C (**b**) of the Co-M (M = Fe, Cr, and Mn) catalysts.

Figure 4a shows the O_2_-TPD profiles of the Co-M (M = Fe, Cr, and Mn) catalysts. The oxygen desorption peak in the temperature range of 50–300 °C corresponds to physically adsorbed oxygen (labeled as α_1_-O_2_), while the desorption peak between 300–500 °C corresponds to chemically adsorbed oxygen species (labeled as α_2_-O_2_). The oxygen desorption peaks above 500 °C belonged to the lattice oxygen species (labeled as β-O_2_) [34]. It was well-established that adsorbed oxygen species played a pivotal role in catalytic oxidation reactions [35]. In Figure 4b, it is evident that the Co-Fe catalyst demonstrates the highest oxygen desorption capacity, peaking at 0.14 mmol·g^−1^ at temperatures below 500 °C. The Co-Cr catalyst exhibits a lower oxygen desorption capacity at 0.11 mmol·g^−1^. Meanwhile, the Co-Mn (0.06 mmol·g^−1^) and CoO*_x_* (0.08 mmol·g^−1^) catalysts displayed the lowest oxygen desorption capacities among the tested samples. Li et al. reported that the mutual doping of various metals induces a shift in the metals towards lower oxidation states, which contributes to an increase in the surface oxygen adsorption content over the catalyst [36].

### 2.3. Catalytic Activity

**Figure 5:**
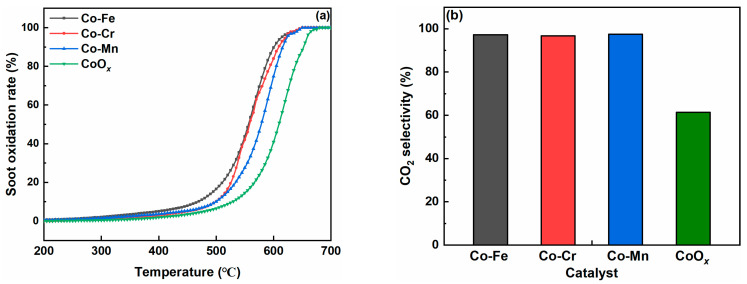
Soot oxidation activity over the Co-M (M = Fe, Cr, and Mn) catalysts in the loose contact mode: (**a**) soot oxidation rate and (**b**) CO_2_ selectivity.

Soot oxidation over the Co-M (M = Fe, Cr, and Mn) catalysts was tested using the TPO method. Figure 5a shows the effect of Co-M (M = Fe, Cr, and Mn) on catalytic soot oxidation in the loose contact mode. It is evident that, regardless of the catalyst type, the soot oxidation rate increases with the increasing reaction temperature. The values of T_10_, T_50_, and T_90_ for the CoO*_x_* catalyst were 527 °C, 608 °C, and 653 °C, respectively. For the Co-Mn catalyst, the T_10_, T_50_, and T_90_ values decreased by 29 °C, 30 °C, and 36 °C, respectively. The Co-Cr catalyst exhibited relatively better catalytic activity with the T_10_, T_50_, and T_90_ values of 497 °C, 558 °C, and 610 °C, respectively. The T_10_, T_50_, and T_90_ values of the Co-Fe catalyst were 470 °C, 557 °C, and 602 °C, respectively, which were 51–57 °C lower than those of the CoO*_x_* catalyst. The T_50_ values for the CoO*_x_* and Co-M (M = Fe, Cr, and Mn) catalysts followed the order of CoO*_x_* (608 °C), Co-Mn (578 °C), Co-Cr (558 °C), and Co-Fe (557 °C). In Figure 5b, the CO_2_ selectivity values of the CoO*_x_* and Co-M (M = Fe, Cr, and Mn) catalysts during the whole reaction process are depicted. It is evident that doping transition metals into the CoO*_x_* catalyst significantly promotes the generation of CO_2_. As shown in Table 3, the Co-Mn catalyst exhibits the highest CO_2_ selectivity of 97.4%, followed by Co-Fe (97.3%), Co-Cr (96.7%), and CoO*_x_* (61.3%). Table 4 shows the comparison of soot oxidation activity among various Co-based catalysts reported in the literature. The T_10_, T_50_, and T_90_ values were significantly improved over Co-based catalysts with distinctive morphologies compared to the Co-Fe catalyst. However, compared with the Co-based catalysts doped with various other components, the Co-Fe catalyst certainly showed relatively better catalytic activity towards soot oxidation.

The crystalline size was determined by the Scherrer equation, as shown in Table 1. The crystallite size of the Co-Fe catalyst was 48.3 nm, which was higher than those of CoO*_x_* (46.2 nm), Co-Mn (25.7 nm), and Co-Cr (24.6 nm). Generally, catalytic soot oxidation mainly occurred on the surfaces of the Co-M catalysts. Thus, the specific surface area and pore diameter were significant factors influencing the catalytic activity. It was well known that a larger specific surface area can provide more active sites for catalytic reactions [40]. The specific surface areas of Co-Fe and Co-Cr catalysts were 9.9 m^2^·g^−1^ and 18.9 m^2^·g^−1^, which were significantly larger than those of Co-Mn (3.7 m^2^·g^−1^) and CoO*_x_* (0.5 m^2^·g^−1^). The pore diameter of the Co-Fe catalyst was 38.6 nm, while the pore diameters of the Co-Cr, Co-Mn, and CoO*_x_* catalysts were 18.9, 15.7, and 9.9 nm, respectively. Since the average particle size of Printex-U soot was 25 nm [41], the larger pore diameter of the Co-Fe catalyst could facilitate the transportation of soot particles and improve their contact with the internal surfaces of the Co-Fe catalyst. Thus, the catalytic activity of soot oxidation over the Co-Fe catalyst was significantly enhanced since the active sites and oxygen species were probably better utilized during soot oxidation.

The catalytic soot oxidation on Co-based catalysts followed the Mars-van Krevelen mechanism, and the abundance of reactive oxygen directly determined the activity of soot oxidation. The primary reaction pathways for catalytic soot oxidation involved reactions in the gas phase and on the catalyst surface. In the initial stages, oxygen in the gas phase preferentially adsorbed onto the oxygen vacancies on the catalyst surfaces. Xu et al. reported that the presence of abundant Co^2+^ on the catalyst’s surface indicated a larger number of structural defects (oxygen vacancies) and better redox properties, thereby promoting the oxidation of soot particles [42]. In this work, the Co-Fe catalyst demonstrated the highest relative concentration of Co^2+^, which consequently resulted in the highest numbers of oxygen vacancies. The XPS spectrum of O 1s revealed that the Co-Fe catalyst also showed a higher concentration of O_ads_ compared to the other catalysts. It was widely recognized that O_ads_ had better mobility than the O_latt_ species, and it could directly participate in soot oxidation through contact points between the catalyst and the soot particles [43]. The O_2_-TPD profile of the Co-Fe catalyst exhibited higher O_2_ desorption levels in the temperature range of 100 °C to 300 °C and the temperature range of 300 °C to 500 °C compared to the other two samples (in Figure 5). Higher O_2_ desorption levels indicated that the Co-Fe catalyst possessed the highest content of reactive oxygen species compared with the other employed catalysts, which could be conducive to accelerating the soot oxidation process. These results are consistent with the results of the XPS profiles.

Finally, the reactive oxygen species experienced adsorption and desorption processes and reacted with soot to generate the final products of CO and CO_2_ [44]. Ji et al. reported that adjacent metal ions in close proximity to oxygen vacancies tended to donate electrons and be oxidized to higher valence states [45]. Subsequently, the adsorbed oxygen species could be converted to O_2_^−^, O^−^, and even O^2−^ species via electron transfer processes [46]. Finally, these activated active oxygen species were desorbed and reacted with the adjacent soot particles to generate CO and CO_2_. As the reaction progressed, oxygen vacancies were then regenerated and the electrons could also be released. The released electrons could be captured by Co^3+^ species, resulting in a reduction from Co^3+^ to Co^2+^, ensuring the redox cycling of the Co metal ions.

### 2.4. Effect of Operating Parameters

#### 2.4.1. Effect of NO

**Figure 6:**
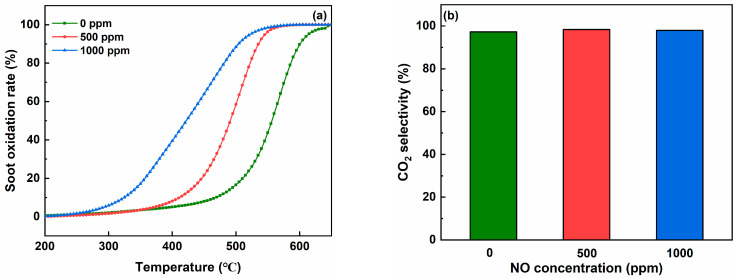
Effect of NO on the soot oxidation over the Co-Fe catalyst in the loose contact mode: (**a**) soot oxidation rate and (**b**) CO_2_ selectivity.

The effect of NO on the catalytic activity of soot oxidation over the Co-Fe catalyst was investigated. Obviously, NO has a significant positive effect on soot oxidation. The values of T_10_, T_50_, and T_90_ were 411 °C, 491 °C, and 535 °C at the NO concentration of 500 ppm, which were lower than 470 °C, 557 °C, and 602 °C without NO addition, respectively. When the NO concentration further increased to 1000 ppm, the values of T_10_, T_50_, and T_90_ decreased further to 378 °C, 451 °C, and 498 °C, respectively. Figure 6b shows the effect of NO addition on the CO_2_ selectivity of soot oxidation over the Co-Fe catalyst. As shown in Table 5, the variation in CO_2_ selectivity was within the range of 97.3% to 98.3% with the increasing NO concentration over the Co-Fe catalyst. NO could be adsorbed by the oxygen vacancies on the catalyst’s surface and oxidized to NO_2_ by reactive oxygen species at the early stages of the catalytic soot reaction. Meanwhile, NO_2_ possesses remarkable oxidizing characteristics, which can directly react with and oxidize soot to CO and CO_2_ [47]. Ranji-Burachaloo et al. also confirmed that the adsorption of NO_2_ onto the surface of the catalyst could greatly facilitate catalytic soot oxidation due to its stronger oxidative property [48].

#### 2.4.2. Comparison between Real and Model Soot

**Figure 7:**
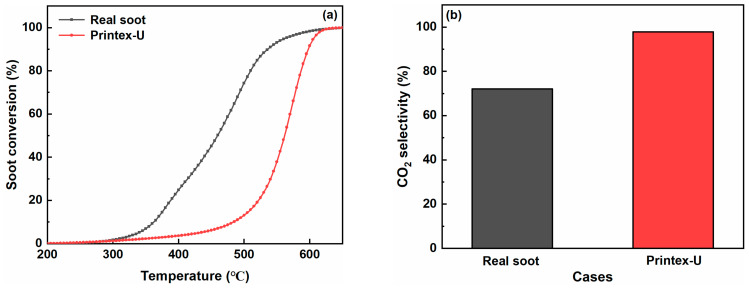
Effect of real soot on the soot oxidation over the Co-Fe catalyst in the loose contact mode: (**a**) soot oxidation rate and (**b**) CO_2_ selectivity.

The microscopic structure and reactivity of Printex-U was quite different from that of real soot, since real soot particles contain soluble organic fractions and various types of metal impurities [49]. Figure 7a indicates that the oxidation process of real soot is much quicker than that of Printex-U since the T_50_ value of real soot is 557 °C, which decreases by 102 °C compared to Printex-U. The improvements could be attributed to the presence of impurities and surface functional groups in the real soot, which potentially provided more active sites, thereby facilitating the progress of the oxidation reaction [50]. Meanwhile, the lower soot content in real soot is a crucial factor leading to its lower CO_2_ selectivity when compared to Printex-U. As shown in Table 6, the CO_2_ selectivity of real soot is only 72.1%.

#### 2.4.3. Effect of Contact Mode

**Figure 8:**
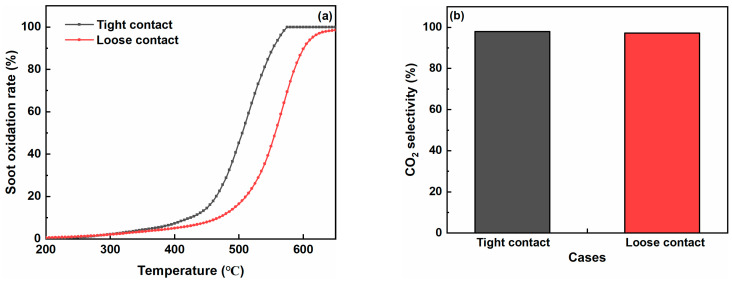
Effect of the contact mode on catalytic soot oxidation over the Co-Fe catalyst: (**a**) soot oxidation rate and (**b**) CO_2_ selectivity.

The contact mode between the catalyst and soot particles also showed a significant effect on the catalytic soot oxidation process. The soot oxidation rate and CO_2_ selectivity were tested over the Co-Fe catalyst in both loose and tight contact modes. The values of T_10_, T_50_, and T_90_ in the tight contact mode were 424 °C, 507 °C, and 552 °C, respectively, which were 46–50 °C lower than the values in the loose contact mode (in Figure 8 and Table 7). The CO_2_ selectivity reached 97.9% in the tight contact mode, which was 0.6% higher than the loose contact mode. The better contact between the catalyst and soot in the tight contact mode can enhance the utilization of active sites on the surface of the Co-Fe catalyst when compared to the loose contact mode. Machida et al. also observed that the adsorbed oxygen species exhibited better transfer efficiency results in the tight contact mode [51]. Moreover, the occurrence of an “O_2_ slip” was potentially reduced in the tight contact mode, leading to a better utilization of released oxygen for catalytic soot oxidation [52].

## 3. Materials and Methods

### 3.1. Materials

Ferric nitrate nonahydrate (Fe(NO_3_)_3_·9H_2_O, AR), chromic nitrate nonahydrate (Cr(NO_3_)_3_·9H_2_O, AR), cobaltous nitrate hexahydrate (Co(NO_3_)_2_·6H_2_O, AR), manganese nitrate solution (50 wt.% Mn(NO_3_)_2_, AR), and anhydrous citric acid (C_6_H_8_O_7_, AR) were all purchased from Aladdin Reagent Co., Ltd., Shanghai, China. All the reagents were used as received without further purification.

### 3.2. Preparation of the Co-M Catalysts

In this study, the Co-M (M = Fe, Cr, and Mn) catalysts were prepared using the citric acid method. Taking the Co-Fe catalyst as an example, 0.01 mol (4.04 g) of Fe(NO_3_)_3_·9H_2_O and 0.01 mol (2.91 g) of Co(NO_3_)_2_·6H_2_O were dissolved in deionized water to obtain a solution (0.4 mol·L^−1^ in metal ions). The solution was then stirred continuously for 30 min. Secondly, the samples were transferred to a water bath and magnetically stirred for 4 h at 90 °C. The mixture was then dried at 110 °C for another 12 h. Finally, the resulting product was calcined at 600 °C for 5 h. The obtained samples were sieved using 40–60 meshes and the catalyst was denoted as Co-Fe.

### 3.3. Catalyst Characterizations

The N_2_ adsorption–desorption of isotherms were performed at −196 °C using an TriStar II 3020 analyzer (Micromeritics, GA, USA). to determine the surface area and average pore diameter distribution. The specific surface areas were calculated using the Brunauer–Emmett–Teller (BET) method. The total pore volume and average pore diameter distribution of the samples were determined using the Barrett–Joyner–Halenda (BJH) method.

X-ray powder diffraction (XRD) patterns were obtained using a D/max-2000 instrument (Rikagu, Tokyo, Japan) with Cu-Kα radiation. The scanning rate of 10°·min^−1^ was employed within the 2θ range of 20–80°. The crystalline size was calculated using the Scherrer’s equation.

To investigate the chemical valence states of the catalysts, an X-ray photoelectron spectroscopy (XPS) analysis was performed using a ThermoEscalab 250Xi system (Thermo Fisher Scientific, Waltham, MA, USA) with Al-Kα radiation. The binding energies were calibrated using the C 1s photoelectron peak at 284.8 eV.

The oxygen temperature programmed desorption (O_2_-TPD) measurements were conducted using an AutochemII 2920 instrument from Micromeritics, Norcross, GA, USA. To remove impurities, 200 mg catalysts were pretreated at 300 °C for 1 h in an He stream (30 mL·min^−1^), and subsequently cooled down to room temperature. Prior to each test, the sample was treated under an O_2_ flow condition at the rate of 30 mL min^−1^ at 70 °C for 1 h. The sample was purged by a flowing pure He stream (30 mL·min^−1^) to remove excessive and weakly adsorbed O_2_. Finally, the sample was heated to 900 °C at a constant heating rate of 10 °C·min^−1^, and the desorption profile of O_2_ was recorded.

### 3.4. Experimental System

The catalytic activities of the Co-M (M = Fe, Cr, and Mn) catalysts were investigated by the temperature-programmed oxidation (TPO) experiment. In this study, Printex-U (Degussa, 20–30 nm) was employed as a surrogate for soot, while the real soot particles were collected from the exhaust of a G6300ZC6B diesel engine (Zhongce Power Electromechanical Group Co., Ltd., Ningbo, China) with 300 mm a cylinder bore, rated power of 1000 kW, and rated speed of 1000 r·min^−1^. 

In each experiment, weighed amounts of catalyst powder (180 mg) and soot particles (20 mg) were mixed in a crucible by gently stirring them with a spatula for 10 min to achieve a loose contact mode. The mixture of the catalyst and soot was packed inside the reactor and held by quartz wool. The simulated gas for the test was composed of 10 vol.% O_2_ and balanced N_2_, while the total flow rate of the simulated gas was 200 mL·min^−1^. The oxygen and nitrogen carrier gases were premixed and then fed into a fixed-bed quartz reactor. In addition, the catalytic oxidation of real soot was investigated under two different conditions (10 vol. % O_2_ + balanced N_2_ and 1000 ppm NO + 10 vol. % O_2_ + balanced N_2_) over the Co-Fe catalyst. The gas cylinders of NO/N_2_, N_2_ and O_2_ were all purchased from Fangxin Gas Co., Ltd., Ningbo, China. The reaction temperature was increased from 50 °C to 700 °C at a heating rate of 5 °C·min^−1^. 

During the experiment, the CO and CO_2_ concentrations in the effluent were monitored and recorded by an infrared gas analyzer with a measurement accuracy of ±3% F.S. (Huayun GXH-3010/3011AE). The temperatures corresponding to 10%, 50%, and 90% soot oxidation rates (denoted as T_10_, T_50_, and T_90_, respectively) were taken as indices of the catalytic activity. The soot oxidation rate (denoted as α) and CO_2_ selectivity (denoted as S_CO2_) were calculated by integrating the CO and CO_2_ concentration profiles with time:

(1) $$\alpha \;\left(\%\right)=\frac{\int_{0}^{t}\left({\left[CO_{2}\right]}_{\mathrm{out}}+{\left[CO\right]}_{\mathrm{out}}\right)\mathrm{dt}}{M}\times 100\%$$

(2) $$S_{CO_{2}}\;\left(\%\right)=\frac{\int_{0}^{t}{\left[\mathrm{CO}\right]}_{\mathrm{out}}\mathrm{dt}}{\int_{0}^{t}\left({\left[CO_{2}\right]}_{\mathrm{out}}+{\left[\mathrm{CO}\right]}_{\mathrm{out}}\right)}\times 100\%$$

where ${\mathrm{CO}}_{\mathrm{out}}$ and ${\mathrm{CO}}_{2}{}_{\mathrm{out}}$ are the outlet concentrations of CO and CO_2_, respectively, while M is the weight of the initially packed soot.

## 4. Conclusions

The catalytic activity of soot oxidation over Co-based catalysts was enhanced by doping CoO*_x_* with various transition metals, including Fe, Cr, and Mn. The soot particles were completely converted into CO and CO_2_ over the four tested catalysts in the temperature range of 50 to 700 °C. The value of T_50_ for the Co-Fe catalyst was 557 °C, which was 1 °C, 21 °C, and 51 °C lower than Co-Cr, Co-Mn, and pure CoO*_x_* catalysts, respectively. The Co-Fe catalyst was also effective for the oxidation of real soot since the T_50_ value was 455 °C, which was notably lower than Printex-U (557 °C). The process of soot oxidation over the Co-Fe catalyst was effectively promoted with 1000 ppm of NO addition, resulting in a decrease in the T_50_ from 557 °C to 451 °C. The soot oxidation process was also significantly enhanced in the tight contact mode since the T_50_ value decreased by 50 °C compared to the loose contact mode.

The XRD results indicate that the Co-M (M = Fe, Cr, and Mn) catalysts maintained an excellent crystalline structure, while the Co-Fe catalyst exhibits the largest specific surface area and pore diameter. The O_2_ desorption amounts and relative concentrations of Co^2+^ and O_ads_ species were closely related to the abundant oxygen vacancies on the surfaces of the Co-M catalysts. During the reaction, the activated oxygen species on the surface was desorbed from oxygen vacancies and subsequently reacted with adjacent soot particles to generate CO and CO_2_. Concurrently, the oxygen vacancies were replenished by oxygen atoms presented in the gas phase. Thus, the numbers of active sites and oxygen species of the Co-M (M = Fe, Cr, and Mn) catalysts were the fundamental factors that influenced the catalyst activity. It is worth noting that the order of the reaction performance is the same with these factors based on the results of the XPS and O_2_-TPD.

## Figures and Tables

**Table 1:** Textural properties of the Co-M (M = Fe, Cr, and Mn) catalysts.

Catalyst	Crystalline Size (nm)	S_BET_ (m^2^·g^−1^)	V_P_ (mm^3^·g^−1^)	D_P_ (nm)
Co-Fe	48.3	9.9	95.3	38.6
Co-Cr	24.6	18.9	89.1	18.9
Co-Mn	25.7	3.7	14.5	15.7
CoO*_x_*	46.2	0.5	1.3	9.9

**Table 2:** The redox properties of the Co-M (M = Fe, Cr, and Mn) catalysts.

Catalyst	O_2_ Consumption (mmol·g^−1^)	Co^2+^/(Co^2+^ + Co^3+^) (%)	O_ads_/(O_ads_ + O_latt_) (%)
Co-Fe	0.14	68.6	51.7
Co-Cr	0.11	60.4	31.2
Co-Mn	0.06	58.6	42.0
CoO*_x_*	0.08	56.6	47.9

**Table 3:** Catalytic activity of the Co-M (M = Fe, Cr, and Mn) catalysts for soot oxidation in the loose contact mode.

Catalyst	T_10_ (°C)	T_50_ (°C)	T_90_ (°C)	S_CO2_ (%)
Co-Fe	470	557	602	97.3
Co-Cr	497	558	610	96.7
Co-Mn	498	578	617	97.4
CoO*_x_*	527	608	653	61.3

**Table 4:** Comparison of soot oxidation activity among Co-based catalysts in the loose contact mode.

Catalysts	T_10_ (°C)	T_50_ (°C)	T_90_ (°C)	References
CoO*_x_* (3D nanostructure)	380	416	448	[17]
NiO-NiCo_2_O_4_ (urchin structure)	348	404	436	[37]
Co-Fe	470	557	602	This work
NiCo_2_O_4_	N.A.	585	626	[38]
CuCo_2_O_4_	N.A.	574	620	
ZnCo_2_O_4_	N.A.	569	602	
Co/KMn	480	604	672	[39]
Co/Cu-KMn	467	610	676	

**Table 5:** Catalytic activity of the Co-Fe catalyst for soot oxidation in the loose contact mode at different NO concentrations.

NO Concentration (ppm)	T_10_ (°C)	T_50_ (°C)	T_90_ (°C)	S_CO2_ (%)
0	470	557	602	97.3
500	411	491	535	98.3
1000	378	451	498	98.0

**Table 6:** Catalytic activity of the Co-Fe catalyst for Printex-U and real soot in the loose contact mode.

Cases	T_10_ (°C)	T_50_ (°C)	T_90_ (°C)	S_CO2_ (%)
Printex-U	470	557	602	97.3
Real soot	357	455	531	72.1

**Table 7:** Catalytic activity of the Co-Fe catalyst for soot oxidation in the loose contact and tight mode.

Contact Mode	T_10_ (°C)	T_50_ (°C)	T_90_ (°C)	S_CO2_ (%)
Loose contact	470	557	602	97.3
Tight contact	424	507	552	97.9

## Data Availability

The data presented in this study are available on request from the corresponding author.
